# Oxidative Stress: Promoter of Allergic Sensitization to Protease Allergens?

**DOI:** 10.3390/ijms18061112

**Published:** 2017-05-23

**Authors:** Leonie S. van Rijt, Lara Utsch, René Lutter, Ronald van Ree

**Affiliations:** 1 Department of Experimental Immunology, Academic Medical Center, University of Amsterdam, 1105 AZ Amsterdam, The Netherlands; Lara.utsch@gmail.com (L.U.); R.Lutter@amc.uva.nl (R.L.); R.vanree@amc.uva.nl (R.v.R.); 2 Department of Respiratory Medicine, Academic Medical Center, University of Amsterdam, 1105 AZ Amsterdam, The Netherlands; 3 Department of Otorhinolaryngology, Academic Medical Center, University of Amsterdam, 1105 AZ Amsterdam, The Netherlands

**Keywords:** reactive oxygen species, oxidative stress, allergic sensitization

## Abstract

Allergies arise from aberrant T helper type 2 responses to allergens. Several respiratory allergens possess proteolytic activity, which has been recognized to act as an adjuvant for the development of a Th2 response. Allergen source-derived proteases can activate the protease-activated receptor-2, have specific effects on immune cells by cleaving cell membrane-bound regulatory molecules, and can disrupt tight junctions. The protease activity can induce a non-allergen-specific inflammatory response in the airways, which will set the stage for an allergen-specific Th2 response. In this review, we will discuss the evidence for the induction of oxidative stress as an underlying mechanism in Th2 sensitization to proteolytic allergens. We will discuss recent data linking the proteolytic activity of an allergen to its potential to induce oxidative stress and how this can facilitate allergic sensitization. Based on experimental data, we propose that a less proficient anti-oxidant response to allergen-induced oxidative stress contributes to the susceptibility to allergic sensitization. Besides the effect of oxidative stress on the immune response, we will also discuss how oxidative stress can increase the immunogenicity of an allergen by chemical modification.

## 1. Introduction

Many airborne allergens, like those from house dust mites (HDM), cockroaches, pollen, cat dander and fungi possess protease activity [1,2]. It has been recognized that this protease activity contributes to sensitization to these allergens. Most proteolytic allergens belong to the class of serine proteases like trypsin, chymotrypsin and collagenolytic-like (like HDM Der p 3, 6 and 9, respectively, American cockroach Per a 10, and several fungal allergens like *Aspergillus fumigatus* Asp f 13 and allergens derived from *Alternaria alternate*). Less common proteases include aspartic protease (German cockroach Bla g 2) and metalloprotease (*Aspergillus fumigatus* Asp f 5) [3]. Although many studies have addressed and clarified the underlying complex mechanisms in the pathogenesis of allergic disorders, how protease activity can spark off allergic sensitization is less clear. The nature of the innate immune response after encountering allergens for the first time, preceding the subsequent adaptive immune response, is likely to be crucial for the development of sensitization to the allergen. There is growing evidence that oxidative stress during allergen encounter may play a role in the allergic sensitization process in different ways, by favoring a T helper 2 cell (Th2) immune response but also by increasing immunogenicity of the allergen [4].

Oxidative stress manifests when the production of free radicals such as reactive oxygen species (ROS) overwhelms cellular anti-oxidant defenses [5]. Reactive oxygen species are partially reduced and highly reactive metabolites of O_2_ that include, amongst others, superoxide (O_2_^−•^), hydrogen peroxide (H_2_O_2_), and hydroxyl radicals (^•^OH) [6]. The mitochondrial respiratory chain is a major source of ROS during homeostasis. The primary function of the respiratory chain is to provide energy to the cell. Produced at limited concentrations under strict control of anti-oxidant mechanisms, they have important roles in cell signaling, growth and homeostasis [5,6]. As an example, when stimulated by cytokines, growth factors and hormones, most cell types elicit a discrete oxidative burst generating low concentrations of ROS [7]. ROS then operate as important messengers of signal transduction through the oxidative modification of kinases and phosphatases, which are present in many signaling pathways including Mitogen-activated protein kinase (MAPK) and NF-κB [7,8]. However, large concentrations of ROS caused by external stimuli or by deficiencies in anti-oxidant systems, may lead to activation of signal transduction pathways and also cause permanent changes in gene expression when these concentrations reach abnormal physiological levels resulting in disease and ultimately cell death [5,9,10,11].

**Figure 1:**
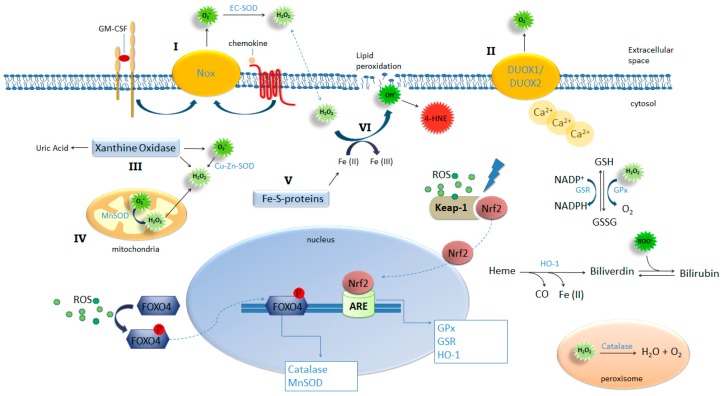
ROS-generating and -detoxifying mechanisms. NADPH oxidases such as Nox-1, -2 and -3 (**I**) and Dual oxidase (DUOX) 1 and -2 (**II**), xanthine oxidase (**III**), mitochondrial respiration (**IV**), and iron-sulfur clusters (**V**) are important sources of ROS production by the cell. Activation of these pathways combined with insufficient or inadequate antioxidant mechanisms result in oxidative stress. NADPH complexes on cellular membrane can be activated by ligand binging on Granulocyte-macrophage colony-stimulating factor (GM-CSF) and chemokine receptors (**I**) and DUOX1 and 2 can be activated by increased intracellular concentrations of calcium (**II**) leading to extracellular generation of superoxide. This radical is dismutated into H_2_O_2_ and oxygen spontaneously or by the action of EC-SOD; Xanthine oxidase can be activated by several pathways including proteolysis and TLR4, triggering generation of uric acid, superoxide and hydrogen peroxide. Increased intracellular production of superoxide results in the release of free irons from iron-sulfur clusters (**V**) feeding Fenton reaction (**VI**) yielding hydroxyl ions. Hydroxyl lipophilic properties result in attack on membrane lipids in a process called lipid peroxidation generating another potent oxidant 4-HNE. Oxidative stress provides the condition for the activation and nuclear translocation of transcription factors FOXO4 and Nrf2 initiating the transcription of key antioxidant enzymes.

ROS generation is generally a cascade of reactions that starts with the production of superoxide. Apart from the mitochondrial respiratory chain, superoxide is generated mainly by xanthine oxidoreductase system (XOR) and NAD(P)H oxidases (NOX) [12,13]. All NOX family members are transmembrane proteins that transport electrons across biological membranes to reduce oxygen to superoxide. The physiological function of NOX enzymes is the generation of ROS for host defense, inflammation and various cellular physiological processes. The majority of NOX enzymes require cytosolic activation or subunit assembly that can be triggered for example, by cytokine and chemokine binding to their respective receptors [12]. Allergen exposure can trigger the release of several chemokines and cytokines, like Granulocyte-macrophage colony-stimulating factor (GM-CSF), by structural or innate immune cells [14,15] and therefore can potentially trigger cytosolic activation of NOX. Dual oxidase (DUOX) 1 and 2 are isoforms of NOX expressed in the airway epithelium and contrary to other NOX, they do not require cytosolic activation or subunit assembly but instead they are directly activated by intracellular Ca^2+^ [12]. Another important source of ROS is XOR. This enzyme system mostly exists in a form of xanthine dehydrogenase (XDH), but it can be converted to xanthine oxidase (XO) by reversible sulfhydryl oxidation or by irreversible proteolytic modification. Oxidation of xanthine by XOR yields uric acid, superoxide and hydrogen peroxide [13]. In addition, free iron released from iron-containing molecules can participate in the Fenton reaction, generating highly reactive hydroxyl (OH^−^) radicals. Therefore heme groups, iron-storage proteins and free iron constitute another potential endogenous source of ROS and oxidative stress [8] (See Figure 1). However, the significance of this reaction is under debate and might only occur in organisms with increased iron levels (as in conditions of hemochromatosis).

The aim of this review is to discuss how oxidative stress induced by proteolytic allergens can contribute to the development of allergic sensitization. There seems to be a delicate balance between the induced reactive oxygen species by exposure to the allergen, for example by activation of protease activated receptor-2, and the anti-oxidant response by the host. We will discuss how allergens with proteolytic activity can disturb this balance and how this can result in allergic sensitization.

## 2. Tissue Damage

The airway epithelium acts as a physical barrier, keeping allergens at the luminal side, but also plays an important immunological role by the release of growth factors, cytokines and chemokines in response to allergens. Disruption of tight junctions can play an important role in the initiation of allergic sensitization. Allergens can cross this barrier more easily and are no longer shielded off from the immune cells underneath the epithelial layer, like dendritic cells. Dendritic cells are important in the differentiation of the immune response and together with the signals supplied by the epithelial cells can skew towards an allergic response. A recent study showed that exposure to HDM induced oxidative damage to proteins, lipids and nucleic acid in lung of mice. Besides disruption of tight junctions, this induced damage could compromise the barrier function of the epithelium [16].

It has been well described that allergen-derived proteases can disrupt the epithelial barrier function by degrading the extracellular domains of tight junction proteins occludin, claudin-1 and E-cadherin [17]. Diffusates from stored pollen of Giant Ragweed, White Birch and Kentucky Blue grass and fresh pollen from Easter Lily were demonstrated to degrade tight junctions in vitro. This could be blocked by protease inhibitors [18]. Also cysteine and serine proteases from pollen [19], HDM Der p 1 [20] and fungi Pen ch 13 [21] have been described to disrupt tight junctions.

Besides direct degradation, HDM proteases are also able to reduce epithelial barrier integrity via PAR-2 activation. Transactivation of the epidermal growth factor receptor destabilizes the E-cadherin protein. Many studies have described that protease allergens are able to induce ROS. The healthy airway epithelium is well equipped with anti-oxidant pathways such as superoxide dismutase catalase, to prevent tissue damage. In asthma, it has been shown that these anti-oxidant responses are suppressed by a defective superoxide dismutase catalase and glutathione peroxidase [22]. The question remains as to whether this is induced by the chronic airway inflammation or whether it is an intrinsic trait of the allergic airways. Interestingly, it has been demonstrated that scavenging superoxide can inhibit the loss of tight junction integrity in type I rat alveolar epithelial cells induced by high-tidal volume mechanical ventilation. In addition, increased expression of Nrf2, a master regulator of the anti-oxidant response, in lung epithelial cells in mice increased the concentration of tight junction proteins zonula and occludens-1 and E-cadherin in the airways. Importantly, in a model in which ovalbumin (OVA) was used as a model allergen, lacking proteolytic activity, pharmacological activation of Nrf-2 by 2-trifluoromethyl-2′-methoxychalone during the allergen challenge was sufficient to reduce allergic inflammation and airway hyperresponsiveness (AHR) [23]. In support of a role for oxidative stress in an OVA-induced asthma model, acute glutathione depletion, suppressing the anti-oxidant response, resulted in an increased airway hyperreactivity and inflammation via p38MAPK-inducible Nitric Oxide Synthase (iNOS) pathway [24]. These experimental data indicate that the induction of oxidative stress after allergen encounter, can favor disruption of tight junctions enhancing the allergic immune response. However, the disruption of tight junctions cannot be attributed exclusively to the protease activity of allergens. A study which compared HDM extracts from different suppliers demonstrated that the extract containing the lowest protease activity caused the most pronounced effects on epithelial barrier function and delocalized E-cadherin the most. This extract evoked the highest release of Chemokine (C-C motif) ligand 20 (CCL20), an important chemokine, and possible other inflammatory mediators which can have contributed to the disrupted epithelial barrier indirectly [25]. This indicates that endogenous proteases, like ADAM metallopeptidase domain 17 (ADAM17), which are induced during inflammation, can increase epithelial permeability and have a similar effect on the disruption of tight junctions. It has been stated before that cockroach allergens and fungal allergens affect the epithelial permeability by the induction of VEGF and TNF-α, respectively by the epithelium [26]. This might also occur via endogenous proteases.

An important alarmin, which is recognized to skew towards a Th2 response, is IL-33. HDM exposure has been described to increase IL-33 production in the airways in HDM allergy models in mice. In addition, bronchoalveolar lavage fluid (BAL) of asthmatics also contained higher levels of IL-33 compared to disease controls [27]. There is some controversy about the release of this cytokine as it is expressed and stored in the nucleus of cells and is known to be released by necrotic cells. Recently, it has been described that the balance between induced oxidative stress by exposure to *Alternaria* extract and the anti-oxidant response plays an important role in controlling IL-33 release by airway epithelium. Human bronchial epithelial cells produced ROS after exposure to *Alternaria* extract, and ROS scavengers were able to prevent extracellular secretion of ATP and suppressed calcium concentrations that precede IL-33 release. In mice administration of a Nrf2 activator, a master regulator of antioxidant machinery, protected against IL-33 release and the allergic immune responses. These data underline the role of oxidative stress in the initiation of the allergic cascade [28]. In addition, there could be an amplifying effect via type 2 innate lymphoid cells secreting IL-13. IL-13 is known to increase mitochondrial ROS [29], which could maintain the disturbed balance resulting in secretion of IL-33.

## 3. Protease Allergens Trigger Reactive Oxygen Species Production in Immune Cells

Certain allergens are able to trigger ROS production and, consequently, oxidative stress. In Vitro it was demonstrated that neutrophils produce ROS after exposure to the major HDM allergens Der-f and Der-f1. Interestingly, the response was increased in neutrophils isolated from HDM-sensitized asthmatic subjects compared with HDM-sensitized non-asthmatic individuals. The ROS production was dependent on the protease activity by Der f because pretreatment with E-64, a cysteine protease inhibitor, eliminated the ROS production [30]. However, also Der p 2 without protease activity or ragweed pollen with intrinsic NADPH oxidase activity were able to induce ROS. The capacity of proteases to induce oxidative stress is well documented [31], but a causal relationship between their capacity to induce oxidative stress and to promote allergic sensitization has only been addressed recently. Tang et al. showed that the cysteine protease papain, when given in combination with OVA, induced an OVA-specific allergic response in mice via the induction of oxidative stress [11]. In our lab, we recently demonstrated that HDM proteases are responsible for the capacity of HDM allergens to induce oxidative stress. Partial inhibition of protease activity dramatically decreased the capacity of HDM to induce oxidative stress and the subsequent allergic inflammation in mice [32].

Csillag et al. demonstrated that treatment with ragweed pollen grains induced maturation of DCs with upregulation of CD80, CD83, CD86 and HLA-DR [33]. The pollen-treated DCs induced the differentiation of naive T lymphocytes toward effector T cells with a mixed profile. Anti-oxidants inhibited both the phenotypic and functional changes of DCs, underlining the importance of oxidative stress in these processes. Goel et al. [34] showed that the cockroach serine protease Per a 10 biased dendritic cells (derived of non-atopic donors) towards a Th2 response by upregulating CD86 while downregulating IL-12. Naïve T cells co-cultured with Per a 10 pulsed DCs showed an increased IL-4 and IL-5 release which was inhibited after inactivation of the proteolytic activity of Per a 10. The ROS production or oxidative stress was not investigated in this experiment. Using a similar approach, we showed that the HDM induced activation of DCs could be inhibited by adding a potent anti-oxidant scavenger *N*-acetyl-l-cysteine during the pulsing with HDM or by heating HDM to eliminate protease activity [32]. These experiments indicate that proteolytic activity induces ROS, which can result in DC activation.

The way in which allergens trigger ROS-generating pathways such as XOR and NOX in host cells is not clear. Proteolytic allergens such as those derived from mites and cockroaches can induce intracellular calcium oscillations through the PAR2-involved pathway [35]. This will be discussed in detail below. Recently, Hristova and colleagues demonstrated that exposure to HDM resulted in an increased DUOX-1 expression in human airway epithelium cells, but not DUOX-2, which correlated with the level of formed H_2_O_2_ and with an increased secretion of the Th2 skewing, innate cytokine IL-33 [36]. In this study, silencing DUOX-1 inhibited secretion of IL-33. The relevance of this mechanism was underlined by the observation that asthmatic patients expressed more DUOX-1 in their nasal epithelium cells after exposure to allergens in comparison to healthy controls, whereas no difference in DUOX-2 expression was found. Allergen exposure leads to uric acid production [37], which initiates XOR activation, however the molecular pathways of XOR activation in this context have not yet been elucidated (see Figure 1).

## 4. PAR-2

Although it has been demonstrated in many studies that allergens with proteolytic activity induce ROS, the underlying mechanism is still unclear. It is important to realize that ROS have important roles in cell signaling. Receptor-mediated signaling often includes the production of ROS, among other functions. Protease activated receptors have an important role in inflammation. PARs are 7-transmembrane G protein coupled receptors that can be cleaved by certain serine proteases. There are four PARs identified of which PAR-2 has the most important role in allergic airway inflammation [38]. PAR-2 is widely distributed on cells in the airways. PAR-2 activation results in extracellular ATP and sustained augmentation of intracellular Ca^2+^ concentrations. Two major allergens of HDM with serine protease activity, Der p 3 and Der p 9, have been demonstrated to cleave the N terminus of PAR-2 at the activation site. Der p 3 and Der p 9 were able to induce the release of GM-CSF and eotaxin in human pulmonary epithelial cells. A PAR-2 agonist was only partially able to desensitize the Der p 3 and p 9 induced cytokine release, indicating that there are alternative pathways involved [39].

PAR-2 activation has been described as disrupting tight junctions between epithelial cells and induces production of growth factors, chemokines and cytokines. This will attract cells from the innate immune system. Mast cells and eosinophils can degranulate after activation of PAR-2 receptors. In addition, airway smooth muscle cells contract and start to proliferate. Trypsin-like protease, which is released by damaged epithelial cells, is a potent activator of PAR-2 [40]. Several allergens possess serine protease activity, like Der p 3 (HDM), *Alternaria* and Per a 10 (cockroach). Proteinase inhibitors were able to reduce allergen-induced airway hyperresponsiveness and inflammation in various animal models for allergic asthma [41,42].

Trypsin has been demonstrated to increase ROS production in isolated lymphocytes in a dose-dependent manner. This was completely inhibited in lymphocytes of PAR-2^−/−^ mice, while PMA and PAF induced a similar concentration dependent production of ROS. This experiment indicates that PAR-2 signaling induces ROS production [43]. In a cockroach-induced allergic airway inflammation model in mice it has been investigated whether PAR-2 induced ROS were crucial in the cascade resulting in allergen sensitization. Challenge with cockroach extract upregulated DUOX-2 and ROS in airway epithelial cells with concomitant increases in airway hyperreactivity and airway inflammation [44]. Reduction of DUOX-2 and ROS by administration of diphenyliodonium attenuated the allergic airway responses, but the attenuation was not as pronounced as that seen after PAR-2 antagonism; indicating that, besides ROS production, other pathways are activated via PAR-2 activation.

In contrast to the demonstrated role of PAR-2 in a mouse model of HDM sensitization [45], Post et al. demonstrated in an experimental HDM allergy model in mice that serine protease is important for allergic sensitization but seems to be largely independent of PAR-2. Post et al. compared two different HDM extracts with different levels of serine protease activity. Only HDM extract containing high levels of serine (and cysteine proteases) was able to induce a HDM-specific IgE response in contrast with an extract with a low level of serine protease activity. In PAR-2 KO mice the HDM-specific IgE response was similar to that observed in wild type mice. Interestingly, the recruitment of eosinophils and Th2 cytokine production was also independent of PAR-2 [46]. This indicates that serine protease activity can contribute to HDM sensitization in a PAR-2 independent pathway.

The type of allergen source plays an important role. In a study in which HDM and *Alternaria* with similar amounts of serine protease were compared for their capacity to induce IL-33 in murine airways, only *Alternaria* was able to induce IL-33 release in murine airways. Blocking serine protease activity or PAR-2 signaling could inhibit this. The early pulmonary cellular infiltrate and the IL-13, but not IL-5, in BALF was dependent on this IL-33 release [1].

## 5. Oxidative Stress Generation Due to Inadequate Antioxidant Responses

In addition to the characteristics of certain allergens to induce ROS, the (in-)capacity to reduce these allergen-induced ROS by antioxidant mechanisms might represent a key role in susceptibility to developing allergic sensitization. Anti-oxidant mechanisms are crucial in the regulation of cellular redox homeostasis. To counteract the deleterious effects of oxidative stress, cells have developed an elaborate anti-oxidant defense to maintain redox equilibrium [47]. Anti-oxidants may be enzymatic or non-enzymatic. Catalase, superoxide dismutase, glutathione peroxidase, glutathione reductase, thioredoxin and heme oxygenase-1 (HO-1) are examples of anti-oxidant enzymes. The non-enzymatic anti-oxidants include glutathione, vitamin E and C, uric acid, albumin, bilirubin and melatonin [48]. Glutathione (GSH) is the major cellular redox buffer contributing to maintaining the intracellular redox homeostasis (see Figure 1). The ratio of reduced and oxidized glutathione (GSH/GSSG) is a good measure of oxidative stress in an organism [8], particularly when ROS are predominantly formed in phagocytes. Deficiency of anti-oxidant mechanisms perturbs intracellular redox status, increasing the basal levels of intracellular ROS affecting cell phenotype and function [33,49,50,51,52,53,54,55,56].

The contribution of inadequate anti-oxidant mechanisms for the development of allergies is slowly being recognized. Polymorphisms in genes involved in ROS detoxification are associated with atopy in humans. The Glutathione S-transferase P1 (GSTP1) gene polymorphic variants are associated with altered catalytic function of this enzyme. The frequency of GSTP1 genotype Val/Val was shown to correlate with lower risk of atopy while the genotypes Ile/Ile and Ile/Val correlated with increased risk of atopy [57,58]. In support, Mapp and colleagues also showed that subjects with occupational asthma to isocyanates were predominantly carriers of the GSTP1 alleles lle/lle or lle/Val while asymptomatic individuals carried the alleles Val/Val [59].

The transcription of anti-oxidant enzyme genes such as catalase and MnSOD are regulated by the forkhead box transcription factor O (FoxO) subfamily, FOXO4 [60,61]; while glutathione reductase (GSR), glutathione peroxidase (GPX), thioredoxin (Trx), thioredoxin reductase (TrxR), and heme oxygenase 1 (HO-1) transcription are regulated by Nuclear factor (erythroid-derived 2)-like 2 (Nrf-2) [62]. In response to oxidative stress, FOXO4 is phosphorylated and subsequently translocates to the nucleus [61]; cytosolic Nrf2 detaches from its inhibitor, Kelch-like ECH-associated protein 1 (Keap 1) and also translocates to the nucleus, where it binds to the Anti-oxidant Response Elements (AREs) [62] (see Figure 1).

The regulation of numerous key anti-oxidant related genes makes Nrf2 a master orchestrator of anti-oxidant responses. The effect of Nrf2 absence in allergic sensitization is well documented. Nrf2 deficiency has been shown to increase the sensitivity to developing allergic sensitization and to promote a Th2 bias phenotype in antigen-presenting cells [53]. In mice, Nrf2 deficiency enhanced the adjuvant effect of ambient ultrafine particles [63,64] and increased susceptibility for the development of a severe airway allergic response to ovalbumin [65]. Un-stimulated Nrf2-deficient cells, contrary to Nrf2 wild type cells, exhibited a pro-type 2 phenotype characterized by a higher baseline level of type 2 related cytokine IL6, and no production of type 1-related cytokine IL12p70 [63].

HO-1, one of the enzymes regulated by Nrf-2 has the capacity to increase the levels of reduced glutathione and to degrade heme into biliverdin, which later turns into bilirubin (see Figure 1). Both sub-products of heme breakdown have potent anti-oxidant properties [66]. HO-1 is expressed at low levels in basal conditions and is rapidly and vigorously induced by oxidative stress and inflammatory stimuli. HO-1 has an essential role in the regulation of inflammation and immune modulation. Both HO-1 knockout mice and human cases of HO-1 gene deficiency exhibit a significantly enhanced pro-inflammatory state [67,68]. HO-1 can modulate inflammation in many ways: inhibition of adhesion molecules on endothelial cells preventing endothelial cells activation and interaction with leukocytes [69]; suppression of neutrophil rolling, adhesion and migration, thereby preventing the entry of neutrophil at the site of inflammation [70]; HO-1 is capable of suppressing the function and proliferation of T effector cells [71]; and when its expression is enhanced on DCs, HO-1 is involved in the induction of CD4^+^CD25^+^ T regulatory cells [72]. Interestingly, compared with normal subjects, eosinophils and neutrophils in asthma patients produced higher levels of H_2_O_2_ and superoxide [73].

## 6. Allergic Sensitization Is Associated with a Decreased Antioxidant Response in Human and in Mice

The relationship between oxidative stress and allergic sensitization is complex because allergen-induced oxidative stress can be at the origin of the Th2 response but, at the same time, inflammation will generate endogenous oxidative stress. We recently observed in our lab that susceptibility for sensitization was associated with an inadequate anti-oxidant response in human. Newly employed animal workers were followed for two years for the development of allergy to rodent urinary proteins [32]. Individuals who became de novo-sensitized to urinary proteins had higher serum levels of 4-HNE modified proteins and a lower expression of HO-1 at baseline before allergen exposure. Both parameters are indicative of a reduced antioxidant capacity. A functional test with PBMCs, which were exposed to X/XO in vitro, showed a lower upregulation of Nrf2 in de novo-sensitized individuals compared with the resistant controls. Although this study underlines that the ability to cope with oxidative stress was a risk factor for the development of allergic sensitization, the underlying mechanisms as to how oxidative stress initiates a Th2 response to allergens remain enigmatic.

To explore this further, we studied the susceptibility to HDM sensitization in a mouse model with two strains of mice, which differed in their capacity to cope with oxidative stress. Sensitization to house dust mite (HDM) in mice was associated with an incapacity to upregulate Nrf2 and HO-1 upon induction of oxidative stress by mite proteases. In BALB/c mice, exposure to a HDM extract with trace levels of endotoxins (LT-HDM) was not sufficient to induce a Th2 immune response [32]. This is in accordance with several studies showing that TLR4 triggering by LPS is crucial for the initiation of allergen-specific Th2 responses IN BALB/c mice [74,75]. However, in mice with a C3H background, the LT-HDM extract induced robust allergic inflammation. Susceptibility of C3H/HeJ to LT-HDM was associated with an inadequate antioxidant response in the lungs after LT-HDM inhalation, while BALB/c resistance to allergic sensitization to LT-HDM was associated with an intact antioxidant response. In support of our finding that a deficient antioxidant system may contribute to allergy development, mouse studies have shown that Nrf2 and HO-1 deficiency predisposes mice to more severe allergic inflammation [65,67,76]. In humans, polymorphisms in genes encoding for enzymes that play a role in scavenging ROS have been associated with their increased risk for the development of atopic airway inflammation, as discussed in the previous paragraph [58].

**Figure 2:**
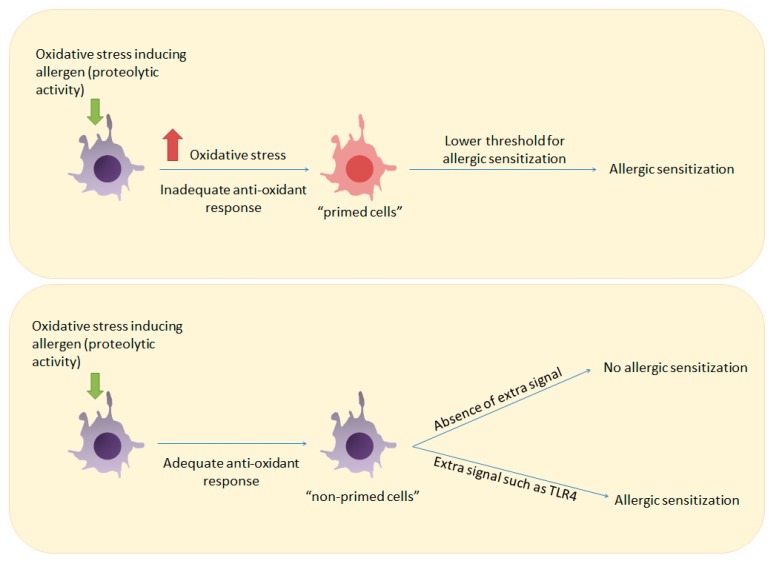
How inadequate anti-oxidant responses can prime the immune system for allergic sensitization. Upper panel: Induction of oxidative stress by an allergen in a host with an inadequate antioxidant response can prime immune cells such as T cells and DCs and lower the threshold for T cell activation. Lower panel: Allergen-induced oxidative stress in a host with an adequate antioxidant response will not result in allergic sensitization unless a stronger signal such as ligation of TLR4 is provided.

Interestingly, we observed a differential threshold for DC maturation in the induction of a Th2 immune response to HDM in these two strains. Intranasal exposure to LT-HDM induced much less maturation of airway DCs compared to a HDM extract containing high levels of endotoxin (HT-HDM) [32,77,78]. Remarkably, C3H/HeJ mice were capable to exert a full-blown allergic type 2 inflammation after intranasal exposure to LT-HDM, despite a low-immunogenic profile of airway DCs (low levels of co-stimulatory surface proteins CD40, CD80, and CD86 compared to HT-HDM). This suggests that a fully mature phenotype of DCs was not necessary for the induction of an allergen-specific Th2 response in C3H/HeJ mice, while in BALB/c this seemed to be a requirement. Because C3H/HeJ showed a defective antioxidant response in the lungs, which was demonstrated by the high level of oxidative stress in lungs at baseline and defective antioxidant protein induction after LT-HDM inhalation [32], we hypothesized that oxidative stress played a key role in the induction of the allergen-specific response to LT-HDM, possibly by decreasing the threshold for T cell activation (see Figure 2).

Although the level of H_2_O_2_ was not determined in DCs and T cells after LT-HDM exposure, preliminary data from our lab showed indeed evidence for H_2_O_2_ as a candidate mediator of allergic sensitization. LT-HDM exposure was followed by an increase in the expression of MnSOD [78] but not of GPx-1 in the lung cells of C3H/HeJ mice. In BALB/c mice an opposite pattern was observed after LT inhalation; no MnSOD induction [78] and enhancement of GPx-1 expression [32]. MnSOD is the main mechanism for H_2_O_2_ generation by the cell while GPx-1 is one of the main systems for H_2_O_2_ removal. Increased MnSOD activity and decreased GPx-1 activity may promote H_2_O_2_ accumulation. This suggests that in C3H/HeJ mice after LT-HDM inhalation generation of H_2_O_2_ was possibly not followed by proper removal and this ROS accumulated. As H_2_O_2_ is able to decrease the threshold for T cell activation, this can compensate for the lack of co-stimulation provided by non-fully mature DC. This hypothesis is in line with the growing body of evidence placing mitochondria, a major generator of physiological H_2_O_2_ via MnSOD [79], as being critical in T cell activation [80].

## 7. Effect of Oxidative Stress on Protein Immunogenicity

An intriguing question related to the causes of the development of allergies is which intrinsic properties of an allergen (physicochemical/biochemical) and which extrinsic factors (e.g., patient-related) make proteins potential allergens. Oxidative stress could play a role in this, both as an intrinsic factor (protein properties promoting oxidative stress and down-stream effects) and an extrinsic factor (anti-oxidant capacity of exposed subject). It is not only inherent characteristics of proteins, which make them capable of inducing oxidative stress (e.g., protease activity or activation of receptors), that are involved; there is also evidence that oxidative stress can enhance the immunogenicity of a protein by tagging it for immune recognition. A study showed that glycolaldehyde is a potential mediator of innate immunity by tagging antigens for immune recognition [81]. Glycolaldehyde is an aldehyde formed after ROS attack on carbohydrates, a process called glycoxidation. Glycolaldehyde introduces aldehydes into protein antigens creating reactive carbonyl moieties that make them more immunogenic. Similar to glycolaldehyde, 4-hydroxy-2-nonenal (HNE) and malondialdehyde (MDA) are among the most common bioactive aldehyde products of oxidative stress [82], generated as a result of a free radical chain breakdown of polyunsaturated fatty acid residues in cholesterol esters, phospholipids, and triglycerides in a process called lipid peroxidation [83,84]. 4-HNE and MDA like glycolaldehyde also modulate proteins by creating reactive carbonyl adducts [85,86]. The occurrence and implication of reactive carbonyl modified proteins in a wide range of oxidation-driven pathologies [87] demonstrate the profound impact of these reactive compounds on the immune response and it is tempting to speculate about their role in allergic sensitization to allergens.

Moghaddam et al. [88] demonstrated that reactive carbonyl adduction on antigens can enhance antigen presentation and T cell proliferation with a Th2 bias, in the absence of conventional co-stimulation. Recently we showed that, as soon as 24 h after inhalation of HDM, 4-HNE reactive carbonyls adducts on proteins could be detected in the lungs of two distinct strains of mice. Interestingly, mice displaying higher levels of 4-HNE protein adducts in lung homogenates both at basal levels and after allergen exposure developed a robust airway allergic inflammation to HDM; while another strain, with lower levels of reactive carbonyl adduction and a better anti-oxidant response, was protected against the development of allergic inflammation [32]. Higher levels of 4-HNE protein modification in these mice indicate an excess in the production of this highly reactive radical. It is possible that allergens administered to these mice in such conditions could undergo aldehyde modifications becoming more allergenic. We were not able to analyze whether the administered allergens were chemically modified by this mechanism. In support of this murine study, atopic individuals with higher basal levels of 4-HNE adducts in serum proteins were also more susceptible to allergic sensitization to a neo-allergen [32]. It would be interesting to investigate whether within the host, in conditions of elevated oxidative stress, allergens will suffer modification by aldehydes, and become (more) immunogenic and therefore increase the likelihood of allergic sensitization.

The development of strategies and technologies to detect exogenous protein “tagging” by oxidative stress by-products in vivo will shed light on the question why antigens are allergens in some individuals but not in others.

## 8. Summary and Concluding Remarks

Oxidative stress induction either through allergens or through deficiencies in key anti-oxidant systems may play an important role in the mechanism of allergic sensitization by affecting immune cell function and phenotype as well as in DC-T cell interaction. In this review, we shed some light on the old question of why a certain protein can cause allergic reactions in some individuals but not in others. Unraveling the ways by which allergens and pollutants trigger ROS-generating systems resulting in oxidative stress and the effects of ROS and other oxidative stress by-products on the immune system will help us design strategies to prevent development of allergies.
